# The establishment and evaluation of a new model for the prediction of Children B-ALL based on TARGET: A SQUIRE-compliant study

**DOI:** 10.1097/MD.0000000000020115

**Published:** 2020-05-08

**Authors:** Xiangyu Gao, Wenjun Liu

**Affiliations:** Department of Pediatrics, Laboratory of Hematologic Tumors and Birth Defects in Children, Affiliated Hospital of Southwest Medical University, Birth Defects Clinical Medical Research Center of Sichuan Province, Luzhou, Sichuan, China.

**Keywords:** acute lymphoblastic leukemia, children, prediction model, TARGET database

## Abstract

B lymphocytic leukemia (B-ALL) is a hematopoietic malignant disease characterized by an accumulation of early B cells. This study aimed to construct a children B-ALL Nomogram prediction model based on Therapeutically Applicable Research to Generate Effective Treatments database, so as to further guide clinical diagnose and treatment.

Clinical data related to children B-ALL were collected from the TARGET database, among which, the stage II clinical data were used as the prediction model, while the stage I clinical data were utilized as the external verification model. The stage II clinical factors were analyzed through Lasso regression analysis to screen the risk factors for the construction of Nomogram prediction model. In addition, the model prediction capacity and accuracy were verified internally and externally using the ROC curve, C-index and calibration curve, respectively.

A total of 1316 B-ALL children were enrolled in this study. Lasso regression analysis revealed that, Age, Gender, WBC, CNSL, MRD29, BMR, CNS R, BCR-ABL1, BMA29, DS, and DI were the important prognostic risk factors. The C-index values of internal and external verification models were 0.870 and 0.827, respectively, revealing the ideal model discriminating capacity. Besides, the calibration curve had high contact ratio, which suggested favorable consistency between the incidence predicted by the model and the actual incidence. Moreover, the AUC values of the ROC curve were 0.858, 0.787, 0.898, and 0.867, respectively, indicating high model prediction accuracy in predicting the 3- and 5-year survival rates of children with B-ALL.The Nomogram prediction model plotted in this study exhibits favorable prediction capacity and clinical practicability for the survival rate of B-ALL children, which contributes to patients screening and clinical intervention.

## 1 Introduction

Acute lymphoblastic leukemia (ALL) is a kind of malignant blood disease induced by the excessive hyperplasia of a certain blood cell system in hemopoietic tissue that invades various tissues and organs. It is characterized by a series of clinical manifestations such as fever, anemia, bleeding, and white blood cell (WBC) invasion. ALL is mostly originated from a single B or T lymphocyte precursor cell, 75% of which are from the B-lymphocyte precursor cells.^[1,2]^ ALL is the first classified using the French American British (FAB) morphological guidelines, which divides ALL into 3 subtypes (L1, L2, and L3) based on cell size, cytoplasm, nucleoli, vacuolation, and basophilia.^[3]^ In 1997, the World Health Organization (WHO) has proposed a comprehensive taxonomy with an attempt to explain the morphological and cytogenetic characteristics of leukemia cells, which identifies three types of ALL: B lymphocytic leukemia (B-ALL), T lymphocytic leukemia (T-ALL) and Burkitt cell leukemia, and B-ALL is the most common malignancy in children.^[3,4]^ The prognosis for children B-ALL is closely correlated with many factors such as age of onset, gender, immune molecules, and cytogenetic abnormality.^[5–7]^ Accurate assessment of prognosis is essential for the treatment of B-ALL.^[3]^ Risk stratification allows physicians to determine the most appropriate initial treatment option and when to consider allogeneic hematopoietic stem cell transplantation (allo-SCT). Traditionally, risk stratification is based on clinical factors, including age, WBC count, and response to chemotherapy, while identification of recurrence and genetic variants will conduce to prognosis assessment and treatment guidance.^[8–10]^ Over the past few decades, the treatment for children B-ALL is markedly improved with the increasing optimization of schemes like chemotherapy and targeted treatment, and the 5-year survival rate has increased to 80% to 90%. However, 15% to 20% children B-ALL cases cannot be relieved for a long time,^[11–13]^ which has threatened children health and brought economic burdens and mental pressure on both the society and the family.

The Therapeutically Applicable Research To Generate Effective Treatments (TARGET) is a public database specific to children tumor, which is managed by the NCI's Office of Cancer Genomic and Cancer Therapy Evaluation Program, and it adopts the multi-omics method to determine the molecular changes in children cancer genesis and development. The major disease items include ALL, acute myeloid leukemia (AML), kidney tumor (KT), neuroblastoma (NBL), and osteosarcoma (OS).^[14]^ Tumor big data provides great assistance for precision medicine developments with stronger specificity and authority, and without data interference.^[15]^ Nomogram is a kind of statistical model constructed for the individualized prediction and analysis of clinical events based on data analysis. It can provide a better individualized prognostic risk assessment in an intuitive and visual way, and offer better prospects for the precision medicine and personalized medicine.

At present, we usually learn the independent prognostic factors for children B-ALL through some previous studies,^[5–7]^ such as Age, Gender and fusion gene, so as to make experimental judgments. However, this can hardly make intuitive and quantitative prognosis prediction analysis, which has restricted the application of these prognostic risk factors in predicting the survival risk for patients during the clinical diagnosis and treatment process. The Nomogram prediction model can well deal with this problem. Currently, intuitive clinical prediction models for multiple cancer types have been constructed based on the databases, such as the Nomogram prediction models for AML and chronic lymphoblastic leukemia (CLL),^[16–18]^ which can favorably predict the survival rate and guide the clinical treatment and prognosis improvement. Nonetheless, there is no prediction model specific to children B-ALL so far. Consequently, this study aimed to construct the Nomogram prediction model based on the TARGET database, which could provide more specific evidence-based medical evidence for its diagnosis and treatment.

## 2 Materials and methods

### 2.1 Data collection

**Figure 1:**
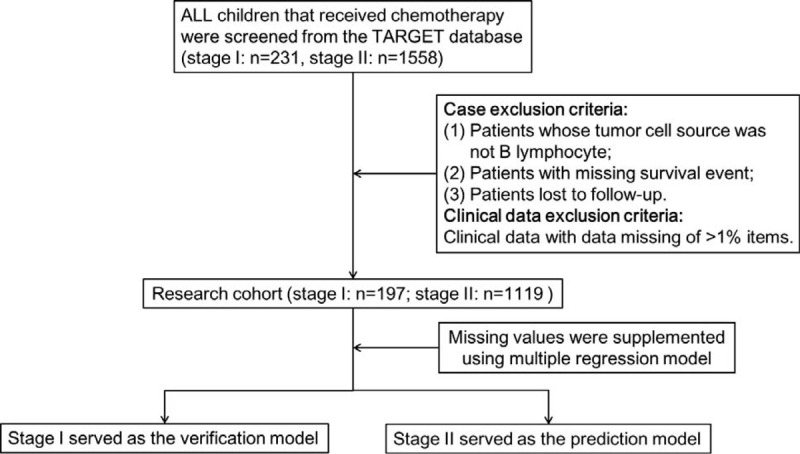
The flow chart of the present study.

B-ALL cases were collected from the TARGET database for Nomogram construction (Fig. 1). The direct deletion of a large number of cases due to the missing of few data should also be avoided, which might result in waste of resources and severe bias in the results.^[15,16]^ Patients were excluded if the follow-up was lost, or any of the survival events data were missing, or more than 1% clinical data were missing. Finally, 197 and 1119 children were enrolled from the stage I and stage II children, respectively. The clinical data included Age, Gender, WBC at initial diagnosis, central nervous system leukemia (CNSL) at initial diagnosis, testicular leukemia, minimal residual disease on day 29 (MRD29), bone marrow relapse (BMR), central nervous system relapse (CNSR), fusion gene BCR-ABL1, bone marrow morphology on day 29 (BMA29), Down syndrome (DS), and DNA index (DI) (Fig. 1). The data in the present study were obtained from the publicly accessible database, which required no ethical review and patient informed consent.

### 2.2 Methods

The variables in the data were classified before modeling according to the children ALL diagnosis and treatment guideline.^[19]^ The basic information was described by constituent ratio using the IBM SSPS-23 software, and the constituent ratio of various variables in two groups of data were analyzed by chi-square test (χ^2^). *P* < .005 was considered as the inspection standard for statistical significance. To avoid direct deletion of some cases due to a small amount of missing data, and avoid the waste of data resources and biased results, R-3.5.1 software was used to run the “VIM” and “mice” software packages to fill up the missing data in stages I and II by the multiple regression method.^[20,21]^ RStudio was employed to screen out the factors that affected the prognosis by Lasso regression analysis on stage I data using the “glmnet” and “survival” software packages. The screen factors were then imported for Nomogram prediction model construction using “rms,” “foreign,” and “survival” software packages for predicting the children survival rate. Then we run the software packages such as “glmnet,” “survival,” and “timeROC,” and the Bootstrap resampling was performed for 1000 times to calculate the C-index, and draw the calibration curve and the receiver operating characteristic (ROC) curve for internal verification of model discrimination, predictability, and accuracy.^[21,22]^ The C-index value ranged from 0 to 1, a value closer to 1 indicated better predictability, while a value of 0.5 represented no predictability, and a value of <0.5 suggested that the predictability was contrary to the real results. The AUC value in the ROC curve of <0.5 suggested no predictability, and the AUC value between 0.51 and 0.7 indicated low accuracy. The AUC value between 0.71 and 0.9 suggested moderate accuracy, and that of 0.9 indicated the high accuracy and high discrimination ability. The coincidence of the calibration curves is related to the accuracy of model prediction, and the higher coincidence indicates more predictive accuracy. The prediction model was externally verified on stage II data by C-index, ROC curve and calibration curve using the RStudio.

## 3 Results

### 3.1 Basic information of patients

The cohort totally included 1119 ALL children (stage II) for modeling and 197 cases (stage I) for verification. Differences in some variables (gender, WBC, and CNSR) between two groups were statistically significant (*P* < .05), whereas those in most variables (Age, CNSL, testicular, MRD29, BMR, BCR-ABL1, BMA29, DS, and DI) were not statistically significant (*P* > .05). Consequently, these results suggested relatively stable distribution of the data variables among the population in the two groups, which could be used for constructing the prediction model (Table 1).

**Table 1:**
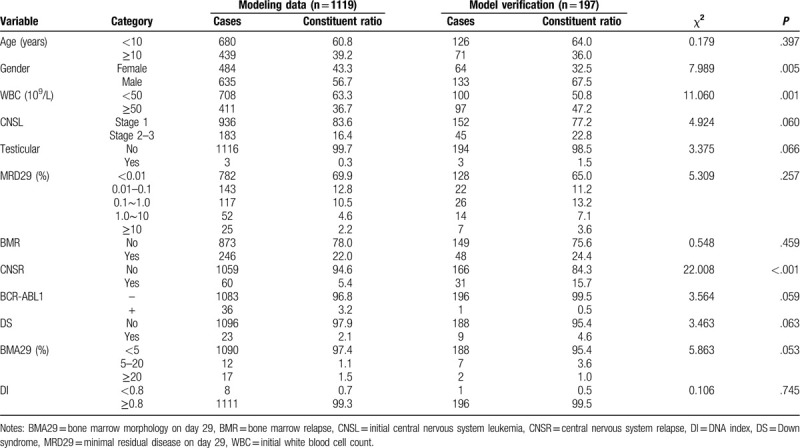
Comparison of basic data and various variables between two groups of children.

### 3.2 Lasso regression analysis

**Figure 2:**
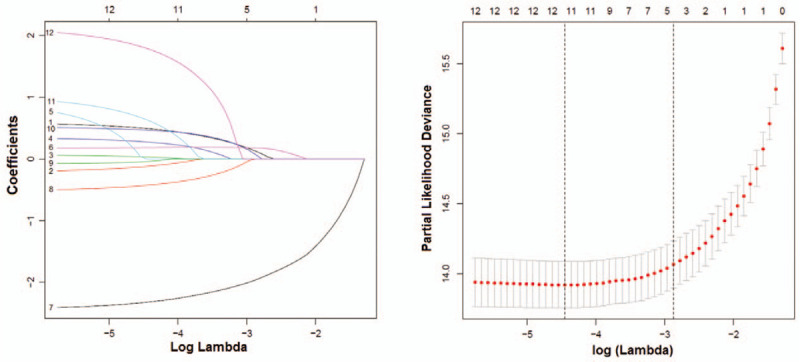
Lasso regression analysis on the survival time of stage II data. (A) Shrinkage estimate graph; (B) generalized cross-validation correction graph, in which the optimal correctional coefficient range lies in between the two dotted lines. 1 represents age, 2 stands for gender, 3 indicates WBC, 4 represents CNSL, 5 denotes testicular, 6 suggests MRD29, 7 reveals BMR, 8 is indicative of CNSR, 9 stands for BCR-ABL1, 10 indicates BMA29, 11 is DS, and 12 stands for DI.

The survival time of stage II data was analyzed by Lasso regression. Figure 2A shows the curve graph after shrinkage estimate of each variable. If the curve regression coefficient tended to be 0, then the variable had poor importance. To take the variable quantity and quality into account, this study selected the dotted line in the left of Figure 2B as the correctional coefficient. Our results suggested that, Age, Gender, WBC, CNSL, MRD29, BMR, CNSR, BCR-ABL1, BMA29, DS, and DI were the important independent risk factors affecting the survival time of children B-ALL, but Testicular was not an influencing factor (Fig. 2).

### 3.3 Construction of the Nomogram prediction model

**Figure 3:**
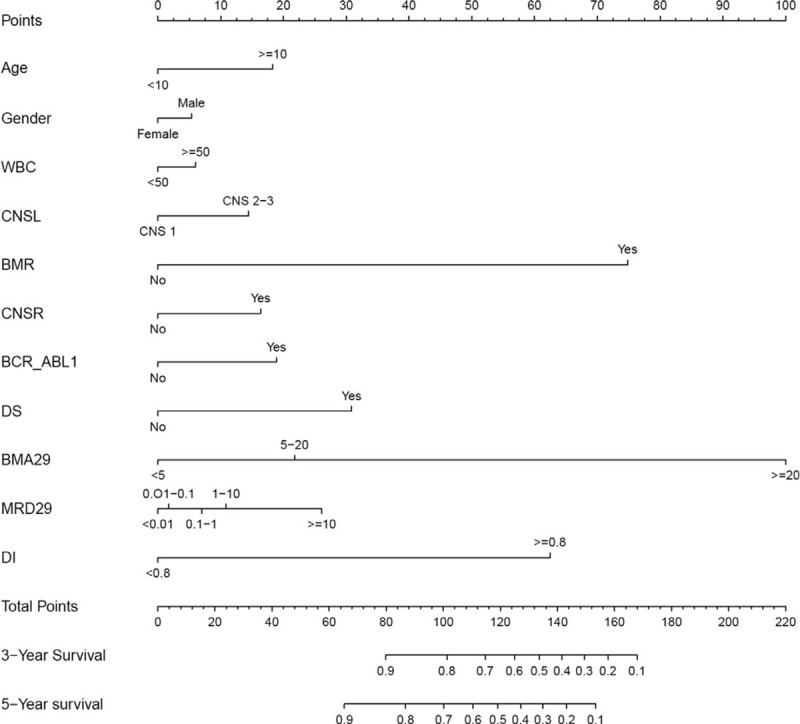
Nomogram prediction model for the 3- and 5-year survival rates for children B-ALL. Points represent the corresponding score of each variable, the Total Points stand for the total score of the survival rate. The corresponding Points of all variables of a child were added to obtain the Total Points, which can be used to obtain the prediction probability of 3- and 5-year survival rates. The precise values are shown in Table 2. A higher total score indicates a lower predicted survival probability.

The important risk factors screened by Lasso regression analysis were imported to construct the Nomogram prediction model, among which, BMA29 and BMR had the highest scores, indicating that they were more important to the prediction results (Fig. 3). The score of each prognosis index was added to obtain the total score, which was then used to calculate the corresponding survival rate for B-ALL patients. If BMA29 > 20% was 100, the score of that patient was 100 if the scores of other factors were 0; so the total score was 100 + 0 = 100. The 3- and 5-year survival rates for B-ALL patients with the total score of 100 were 80% and 70%, respectively (Table 2).

**Table 2:**
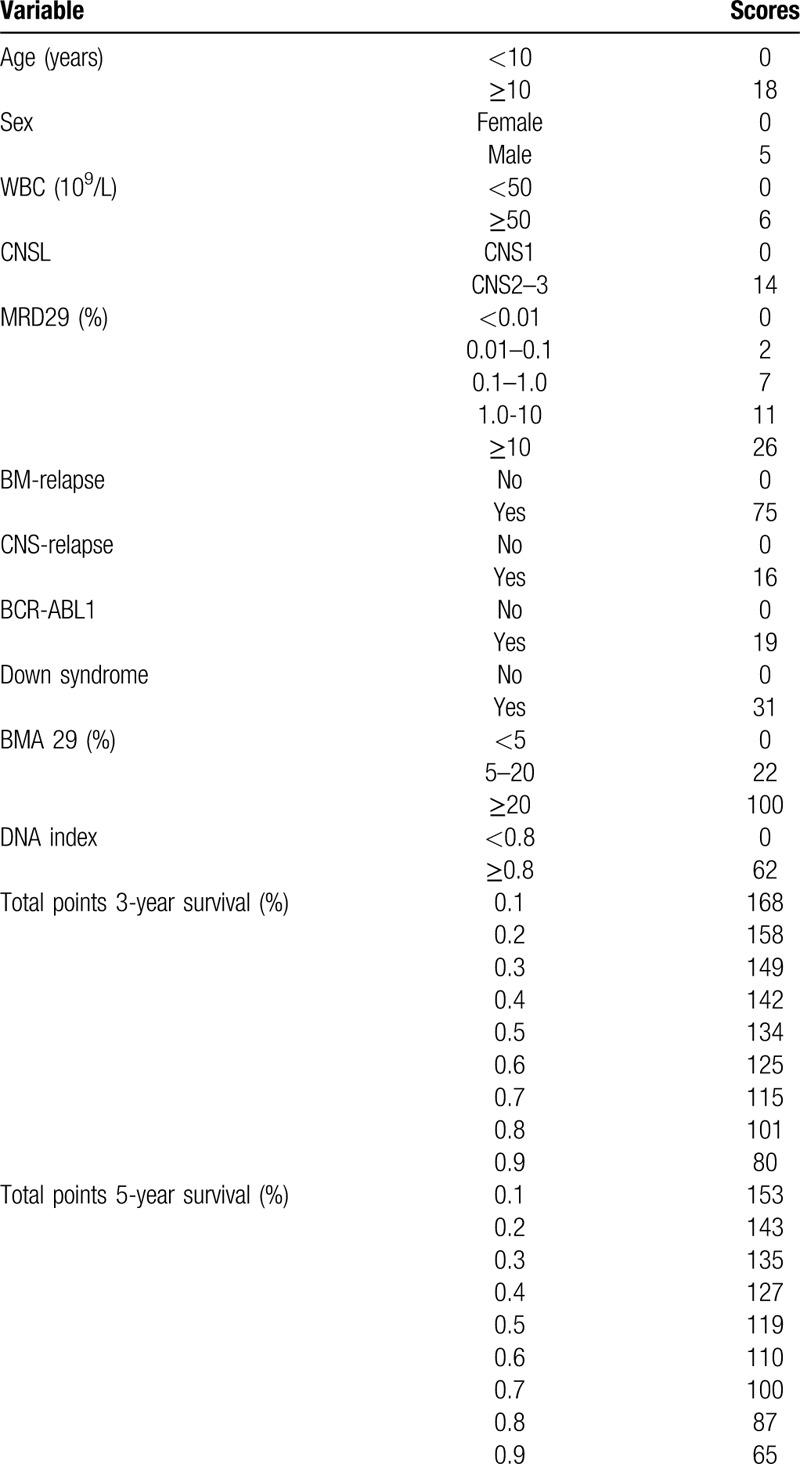
Precise value of each variable in the Nomogram prediction model.

### 3.4 Calculation of C-index and plotting of model calibration graph

**Figure 4:**
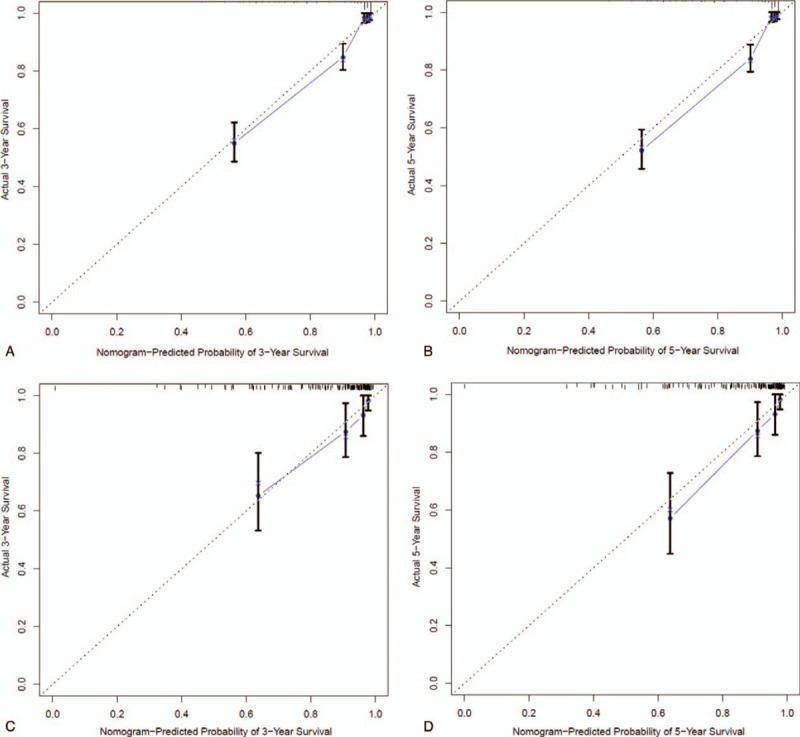
The model calibration graphs were plotted through 1000 times of Bootstrap re-sampling. (A) Calibration curve for internal verification of the 3-year survival rate; (B) Calibration curve for internal verification of the 5-year survival rate; (C) Calibration curve for external verification of the 3-year survival rate; (D) Calibration curve for external verification of the 5-year survival rate. Notes: The dotted lines represent the actual curves; the full lines represent the predicted curve. The actual and predicted curves had high superposition degree, suggesting superior predictability of the prediction model.

The model predictability was verified internally and externally through 1000 times of Bootstrap re-sampling. The C-index values of internal and external verification were 0.870 (standard error of 0.011) and 0.827 (standard error of 0.025), respectively, displaying ideal model predictability. Further external and internal verification as well as plotting of calibration curve revealed that, the 3- and 5-year survival rate prediction curves for children B-ALL were close to the reference curve, which exhibited favorable discrimination and predictability (Fig. 4).

### 3.5 ROC curve

**Figure 5:**
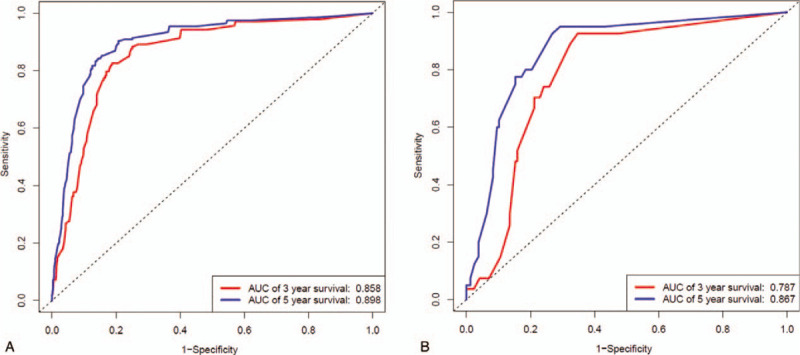
The accuracy of the Nomogram prediction model was verified by the ROC curve. (A) ROC curve for internal verification; (B) ROC curve for external verification. Notes: AUC represents the area under the curve, and a greater AUC value suggests a higher accuracy of the prediction model.

The accuracy of the Nomogram prediction model was verified by the ROC curve. Upon internal and external verification, the AUC values of 3-year survival rates were 0.858 and 0.787, respectively. However, those of 5-year survival rates were 0.898 and 0.867, separately. All these values lied in between 0.71 and 0.9, indicating moderate prediction accuracy of that model (Fig. 5).

## 4 Discussion

As the medical research model evolves from empirical medicine to evidence-based medicine, and to precision medicine, the value of data has never been more important. Nomogram integrates multiple different clinical variables data to establish relevant prediction models to provide a more accurate and personalized prognosis evaluation system to serve the clinic.^[23,24]^ Previous studies have highlighted that the prediction accuracy of Nomogram model is much more higher than the conventional staging systems in different kinds of cancers.^[25–27]^ In this study, the Nomogram prediction model was plotted based on the TARGET database, which was used to predict the 3- and 5-year survival rates of children B-ALL. Besides, the AUC values obtained by internal and external verification were high (0.71–0.9), and the C-index values were >0.8, which revealed excellent consistency and high predictability. In addition, the calibration curve basically coincided, revealing high accuracy of the prediction model, which had higher predictability and accuracy than the prediction models constructed by Chen Cun te, Molica Stefano et al.^[16,17]^ Consequently, the Nomogram prediction model can provide a favorable prognosis assessment system for children B-ALL patients.

Over the past few decades, the risk factors related to the poor prognosis for children ALL are categorized by their clinical risk degree, and treatment schemes with various intensities are adopted based on the clinical risk degree classification, which markedly improve the survival rate of these children. However, the survival rate remains low for children with no bone marrow morphological remission after induced treatment, and the long-term survival rate after chemotherapy can be remarkably increased if children B-ALL can achieve the induced remission status after induced chemotherapy (namely, BMA29 < 5%).^[7,12]^ BMA29 is an index to evaluate the remission induction degree at the end of chemotherapy, and it is an important indicator to judge whether ALL can obtain long-term survival. It is suggested that the recurrence of children B-ALL is mostly bone marrow relapse (BMR) alone. Children with BMR have the worst prognosis, and the worse prognosis is always accompanying with earlier onset of BMR, which is an important independent risk factor affecting prognosis.^[28,29]^ Consistent with precious studies, our present study found that, BMA29 and BMR had high scores in the constructed Nomogram prediction model, suggesting that the two were markedly correlated with the poor prognosis for children B-ALL, which were the most potent indexes in the model to predict the prognosis. Clinicians should pay attention to the selection of B-ALL treatment scheme for those children, and the general characteristics of those children should also be further investigated, so as to provide more references for the new treatment scheme.

Lasso regression analysis in this study demonstrated that, Age, Gender, WBC, CNS, MRD29, BMR, CNSR, BCR-ABL1, BMA29, DS, and DI were the important risk factors affecting the prognosis for children B-ALL, which were consistent with previous studies.^[30]^ It is found that, the newly diagnosed juvenile patients (10–18 years old) with the WBC count of more than 50 × 10^9^/L always have a poor prognosis and BMR, and it is very difficult to treat such cases due to the poor prognosis.^[31]^ However, in the Nomogram prediction model, risk factors including Age, Gender, WBC, and CNS had lower scores than BMA29 and BMR, indicating their poorer predictability. In order to reduce the death risk due to the excessive high-intensity chemotherapy risk or low-intensity chemotherapy induced poor results, more attention should be paid to BMA29 and BMR, and individualized treatment plan should also be developed. Nonetheless, the population-based data in the TARGET database do not include the detailed clinical data, more adverse fusion gene and risk stratification. Recent studies find that, the cytokines related to B-ALL progression, such as granulocyte colony- stimulating factor (G-CSF) that induces progenitor cells proliferation and differentiation to neutrophils, play a crucial role in malignant transformation of leukemia.^[9,10]^ Consequently, more clinical data are necessary to construct and verify the model accuracy and repeatability.

Nevertheless, some limitations should be noted in this study. First, this study was a retrospective study, and different patients had adopted schemes with different intensities, which might lead to bias in the results. Secondly, some missing data in this study were filled through the multiple regression models, and there still existed data deviation. Thirdly, limited variables were enrolled, and multi-center prospective studies should be carried out to further verify and optimize the model.

In summary, the Nomogram prediction model constructed in this study has favorable predictability, which can be used to guide clinical treatment and predict prognosis. However, the sample size should be further enlarged, and more prognosis-related factors should be identified to optimize the model. In addition, our results suggest that BMA29 and BMR are the important risk factors affecting prognosis. Clinicians should pay more attention to such children, seek for better treatment scheme and improve their survival rate.

## Author contributions

**Conceptualization:** Wenjun Liu.

**Data curation:** Xiangyu Gao.

**Formal analysis:** Xiangyu Gao.

**Funding acquisition:** Wenjun Liu.

**Investigation:** Xiangyu Gao.

**Methodology:** Xiangyu Gao, Wenjun Liu.

**Resources:** Xiangyu Gao, Wenjun Liu.

**Software:** Xiangyu Gao.

**Supervision:** Wenjun Liu.

**Writing – original draft:** Wenjun Liu.

**Writing – review & editing:** Wenjun Liu.
